# fNIRS neuroimaging in olfactory research: A systematic literature review

**DOI:** 10.3389/fnbeh.2022.1040719

**Published:** 2022-12-22

**Authors:** Natalie Gunasekara, Giuliano Gaeta, Andrew Levy, Eleanor Boot, Ilias Tachtsidis

**Affiliations:** ^1^Department of Medical Physics and Biomedical Engineering, University College London, London, United Kingdom; ^2^Health and Well-being Centre of Excellence, Givaudan UK Limited, Ashford, United Kingdom; ^3^MetaboLight Ltd., London, United Kingdom; ^4^Wellcome Centre for Human Neuroimaging, University College London, London, United Kingdom

**Keywords:** odor, olfaction, fragrance, essential oils, functional near-infrared spectroscopy (fNIRS), aromatherapy

## Abstract

There are a number of key features which make olfaction difficult to study; subjective processes of odor detection, discrimination and identification, and individualistic odor hedonic perception and associated odor memories. In this systematic review we explore the role functional near-infrared spectroscopy (fNIRS) has played in understanding olfactory perception in humans. fNIRS is an optical neuroimaging technique able to measure changes in brain hemodynamics and oxygenation related to neural electrical activity. Adhering to PRISMA guidelines, results of this search found that generally the majority of studies involving healthy adult subjects observed increased activity in response to odors. Other population types were also observed, such as infants, individuals with autism, attention deficit hyperactivity disorder (ADHD), post-traumatic stress disorder (PTSD), mild cognitive impairment (MCI) and dysosmia. fNIRS coverage heavily favored the prefrontal cortex, temporal and parietal regions. This review finds that odor induced cortical activation is dependent on multiple factors, such as odorant type, gender and population type. This review also finds that there is room for improvement in areas such as participant diversity, use of wearable fNIRS systems, physiological monitoring and multi-distance channels.

## Introduction

The roles of olfaction are diverse and numerous. Living creatures are able utilize smell to evaluate the environment, locate mates and food, and cement familial bonds. Though these are examples of innate uses of smell, odor has a long history of human use. Plague doctors in Europe used lavender pouches in their masks, believing that these fragrant smells would deter disease. Incense has been used for thousands of years during religious ceremonies and prayer throughout the world (Smith, 2018). Similarly, aromatherapy has been used as a treatment for ailments of both mind and body for some time (Sowndhararajan and Kim, 2016). More recently, science and industry have taken an interest in investigating the psychophysiological benefits of plant extracts, perfumes, and essential oils to promote wellbeing, relaxation, and improve sleep quality.

Like sound, odors are capable of traversing great distances, and are highly emotionally influential stimuli. The additional benefit of applicability to objects in our everyday environment lends odor to be highly adaptable asset for promoting changes in mood and state. Using fragrances in items to aid relaxation has become a commonplace occurrence. Aside from the long-time use of incense, candles and essential oils, various household items, such as fabric detergent and home fresheners are utilizing the relaxing effects of odor for both marketing and wellbeing purposes. For example, cleaning products tend to utilize citrus essences such as orange and lemon, as these odors are very often associated with feelings of cleanliness and sanitation (Holland et al., 2005). Odors such as lavender and rose are notable for their relaxant and sedative properties, and are commonly used in cosmetic and laundry products, lending odor to be accessible on-the-go and crucially, during sleep (Field et al., 2005; Kiecolt-Glaser et al., 2008; Sowndhararajan and Kim, 2016).

### Physiological data in olfaction studies

Many studies have observed the effects of odors, particularly essential oils, on mood and relaxation. These effects are also supported by physiological data. Some studies have found that lavender decreases heart rate and skin conductance, whereas a stimulating fragrance such as neroli has the opposite effect (Sowndhararajan and Kim, 2016). Physiological changes induced by lavender included decreased skin conductance, increased blood flow and decreased blood pressure (Saeki and Shiohara, 2001). The distinctive odors of Hinoki and Meniki essential oils studied by Chen et al. (2015) found increased pleasant mood after inhalation. The effect of these odors on relaxation was noted by decreased systolic blood pressure and heart rate. Ylang ylang essential oil inhalation has also been found to increase relaxation and improve mood, as demonstrated by decreased heart rate and blood pressure (Jung et al., 2013). Conversely, some olfactants are understood to increase blood pressure, respiratory rate and heart rate, such as rosemary (Sayorwan, 2013). Rosemary has been found to be a stimulant, improving memory and attention, whilst relaxant naturals such as lavender caused the opposite effect (Moss et al., 2003). Cortisol is a measure of stress and can be modulated by the presence of an anxiolytic fragrance. In a study involving university students during their exam period, Rose essential oil inhalation was found to modulate cortisol levels, as well as skin conductance (Fukada et al., 2011).

Although studies have shown psychophysiological and behavioral changes due to odor, the exact mechanisms of odor action are not well-understood (Kiecolt-Glaser et al., 2008). Despite being a fundamental contributor to so many facets of daily life, the sense of smell is largely overlooked, both generally and scientifically. This may be due to difficulties with odor delivery, the subjectivity of olfactory experiences, and an inability to describe or evaluate odors consistently.

Functional near-infrared spectroscopy (fNIRS) is a relative newcomer to brain imaging and odor research. fNIRS, like fMRI, investigates cerebral blood hemodynamics rather than neural electrical activity. Utilizing optical properties of near-infrared light, fNIRS is able to measure changes in brain activity *via* a system known as neurovascular coupling. Neurovascular coupling is a term used to describe the relationship between neural electrical activity and the changes in cerebral blood flow and oxygenation. In summary, metabolic demand in a particular area increases when neurons in that region become active. As a result, the concentration of oxygenated hemoglobin in the blood around this region is depleted as oxygen is used during neural metabolism. Simultaneously, deoxygenated hemoglobin in the region increases (Chen et al., 2020). To compensate for reduced oxygenation, blood flow to this region increases, causing a reversal in relative chromophore concentrations. At this stage, there is greater oxygenated hemoglobin relative to the deoxygenated hemoglobin in the area. Both chromophores possess differing near-infrared light absorption spectra (NIR) (650–900 nm), enabling the use of NIR light to determine relative concentrations of each. Additionally, the absorption spectra for NIR light has been found to be minimally absorbed by water, bone and tissue (e.g., skin, scalp), allowing NIR light to reach the cortex relatively unimpeded. Utilizing these characteristics and conversion equations such as the Beer-Lambert Law, fNIRS is able to measure relative changes in blood oxygenation and derive regions of neural activity. A comprehensive description of fNIRS principles is beyond the scope of this review. For full coverage of fNIRS, please see the fNIRS publication by Pinti et al. (2020) and Neurophotonics fNIRS publication by Ayaz et al. (2022). From now onwards, the concentrations of oxygenated and deoxygenation hemoglobin in the brain will be referred to as fNIRS measurements. fNIRS devices consist of sources producing NIR light at two or more wavelengths, and detectors receiving NIR light. Based on the proportion of light received by the detectors, and the differing absorption spectra of oxyhaemoglobin and deoxyhaemoglobin at both wavelengths, changes in brain activity are determined.

Comparatively, fNIRS has greater motion artifact resistance than EEG and fMRI, allowing for less restrictive and more versatile studies. If interested in the relaxant effects of a fragrance, the non-invasive, quiet, small size, and portable features of fNIRS are highly suitable. The additional development of wearable fNIRS systems can add significant scope to odor related in-context studies. Although fNIRS has many beneficial features, its disadvantages should be carefully considered before opting for this modality. Similar to fMRI, fNIRS does not directly measure neural electrical activity, instead calculates activity *via* changes in hemodynamic oxygenation. The coupling between neural activity and hemodynamic activity is known to have a slight delay, therefore extra care should be taken to determine task and analysis properties. The predominant disadvantage of fNIRS is low spatial resolution. fNIRS optodes at the standard distance of 3 cm apart are only able to image the cortex to a depth of roughly 5–8 mm (Karim et al., 2012). If measurement of regions that lie deep within the brain are required, fNIRS will likely not be suitable. fNIRS has a lot to offer in other aspects, such as free movement and real-world applications. With populations that may have difficulties remaining still (infants, autism spectrum), fNIRS is an ideal alternative, providing non-invasive, lightweight measurement.

### Aims

Currently, the uses of fNIRS in odor research are highly varied, as is processing and analysis of fNIRS data. This literature review aims to summarize all applications of fNIRS in odor research, and will cover (1) the various protocols used and key experimental features such as fNIRS equipment, channel density, channel location, odourant type, and hemodynamic response to odors. (2) The preprocessing and analysis protocols currently used for odor based fNIRS data. (3) Areas that could benefit from further study, with recommendations for future research. This review will be separated by population type: infants, atypical and healthy control participants, respectively. Through this analysis, readers will be able to understand how fNIRS has been used in odor based research for different populations, odor types, olfactory delivery devices, and paradigms.

## Methods

**Figure 1:**
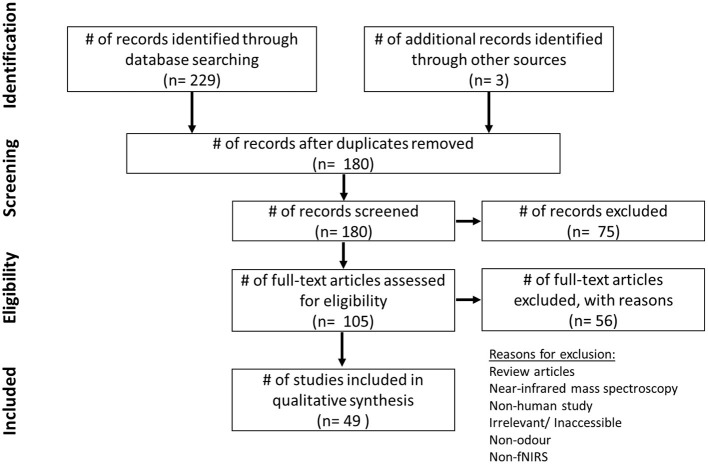
PRISMA chart depicting literature review screening process, including number of papers found with keyword search, papers excluded and retained.

The aim of this systematic review is to summarize the various methods and instruments used to research odour with fNIRS, and compile findings related to the effect of odour on brain activity. Other modalities that were generated *via* the keyword combination were not included if fNIRS was not used. Papers were identified using Web of Science and PubMed using keywords (fNIRS, functional near-infrared spectroscopy; NIRS, fragrance, odor, olfaction, essential oils, smell, aromatherapy). Boolean operators “AND” and “OR” were used to develop combinations for the search string. The PRISMA method was used to search for literature (Figure 1). The literature search for this review was completed in July 2022. Details regarding author, publishing year, odors used, fNIRS device, channel number, region of interest (ROI), and analysis method were extracted and compiled by NG. Due to the inclusion of NIRS, a substantial number of papers were returned based on near-infrared mass spectroscopy. These were immediately removed. Any studies not involving odor, or an aspect of odor were also discarded.

## Results

Using the keywords and databases previously mentioned, 229 results in total were returned in Web of Science and PubMed, between the years 2000–2022. Three additional papers were identified from other sources. Due to overlap between databases, 52 papers were removed. Seventy-five papers were then screened at the initial stage using the inclusion/exclusion criteria, and subsequently excluded from the total. Two hundred twenty-nine papers were filtered through to remove 61 mass spectrometry based papers due to the keyword “NIRS.” Reasons for exclusion can be seen in Figure 1. With more in-depth reading, 56 further papers were excluded in the next stage. The 49 remaining results of this review are presented in Table 1.

**Table 1:** Literature review table summarizing fNIRS studies involving odor stimuli.

**References**	**Participants**	**Instrumentation**	**fNIRS channels**	**Areas of interest**	**Protocol**	**Stimuli**	**HbO2**	**HbR**	**HbT**
Yamashita et al. (2022)	5 (no age info)	fNIRS Hb133, Astem Co., Ltd.	2	OFC	Wearable device. Cross-modal experiment, between scent and light color harmony	Vanilla, mint, lime, grapefruit, cypress	**↑**	Not reported	Not reported
Xu et al. (2020)	•25 autistic, 20.5 years •16 typical, 21.33 years	ETG-7000, Hitachi medical	22	OFC	Triangle odor test on autistic participants of differing detection thresholds. Odor pulse ejection delivery system used	Mint, rose	**↑** Controls	Not reported	Not reported
Casillas-Figueroa et al. (2020)	42, mean 29.43	NIRScout, NIRx USA	6	PFC	HbO2 changes manual vs. ReminiScentia (delivery system)	Lemonade, chocolate mint	**↑**	Not reported	Not reported
Matsumoto et al. (2012)	10, 30.5 ± 4.6 years	ETG-4000, Hitachi Medical	52	Frontal, temporal	Test of odor vs. odorless broth	Bonito flakes flavor/odor, odorless broth	**↑**	Not reported	Not reported
Eto et al. (2016)	15, >65 years	ETG-4000, Hitachi Medical	44	PFC	Foot massage with and without aroma of essential oil	Blend: Lavandula angustifolia, tea tree, Ravensara, Palmarosa, Chamomile	**↓**	Not reported	Not reported
Moein et al. (2020)	17, mean 28.5 ± 6	In-house build TD-fNIRS	4		Odor detection	Garlic, strawberry, water	**↑**	Not reported	Not reported
Frie et al. (2019)	45 (3 groups: 37–41, 33–36, 28–32 weeks)	NIRScout, NIRx USA	4	Olfactory cortices	Infant study during active sleep, odor presentation	Maternal odor	**↑**	Not reported	Not reported
Invitto et al. (2019)	10, mean 25 ± 5.2	NIRScout, NIRx USA	16	PFC & parietal	Haptic study using 3D models and edible/non-edible odors	Grass, rose, mint, lemon, cinnamon	**↓**	No change	Not reported
Song et al. (2019)	21, mean 21.1 ± 1.0	TRS-20 system, Hamamatsu photonics	Not specified	PFC	Hinoki image, blank with Hinoki oil, Hinoki image with oil, blank no oil	Hinoki tree oils	↓ Odor & Audio	Not reported	Not reported
Takakura et al. (2011)	10, 30.4 ± 2.6	NIRSTATION: Shimadzu Co	32	PFC	TPD used as an odourant, but injected. Causes garlic odor sensation	TPD, saline	**↑**	Not reported	Not reported
Muelbert et al. (2021)	5 (±2 days) 10 (±2 days)	NIRO-200, Hamamatsu, Japan	2	PFC	Intervention group shown smell and taste of milk before gastric feed. Control with no exposure	Milk odor, breast, or formula	No change	Not reported	Not reported
Eto et al. (2015)	11, >65	ETG-4000, Hitachi Medical	44	PFC	Massages with and without essential oil aroma	Lavandula angustifolia, tea tree, Ravensara, Palmarosa, and Chamomile	**↓**	Not reported	Not reported
Nishitani et al. (2014)	38, no age info	OM-220, Shimadzu	Not specified	PFC	Mothers and non-mothers presented with infant and adult male odors	Infant and adult male odor	**↑** Mothers	Not reported	Not reported
Hucke et al. (2018)	26 (2 groups) mean 1 (26.43 ± 3.52) 2 (25.08 ± 4.30)	ETG-4000, Hitachi Medical	24	Olfactory. Frontal, middle frontal gyrus	Participants exposed to irritant odors, effect on brain activity measured	acetic acid, ethyl acetate, air (control)	**↑** Acetic acid	Not reported	Not reported
Frie et al. (2017)	41, 28–41 weeks	NIRscout, NIRx USA	8	Olfactory, frontal, somato-sensory	Infants were presented with 3 odorous substances and the control when in active sleep	Pure hand cleaner, diluted hand cleaner, adhesive remover	**↑**	No change	Not reported
Tanida et al. (2008)	12 (no age info)	NIRO-300, Hamamatsu photonics	Not specified	PFC	[Fragrance exposure for 4 weeks. HbO2] changes during mental arithmetic task and sebum secretion measured before and after intervention	Floral green tone spray	**↓**	Not reported	Not reported
Azuma et al. (2016)	16 (no age info)	LABNIRS, Shimadzu	52	PFC	Odor detection and recognition threshold testing of multiple chemical sensitivity cohort	Rose, caramel, sweat, fruit, vegetable, sweet, fecal	**↑**	Not reported	Not reported
Tomono et al. (2021)	10 (21–22 years)	WOT-100 by NeU Corporation	16	PFC, temporal	Food videos presented to ppts *via* a integrated scent dispersal display	Sukiyaki sauce	Varied	Not reported	Not reported
Onuma et al. (2018)	44 (4 groups) mean ages (1) 20.3, (2a) 21.6, (2b), 21.3, (3) 21.3	FOIRE-3000 WOT-S20 Hitachi parotid glands	34	Temporal	Participants tasted NaCl with and without MSG or soy sauce odor	Soy sauce	**↑**	Not reported	Not reported
Rajagoplan (2018)	6, 21–22	HOT-1000, NeuSystems Inc	2	Frontal	Multiplication and word generation tasks were performed before and after drink intake. One drink was used per day	Green tea, transparent sugar milk, lemon teas, water	Not reported	Not reported	Not reported
Gramlich et al. (2017)	48, mean 32.02 ± 8.47	NIRSport-88, NIRx	22	PFC	fNIRS was recorded on combat veterans, combat veterans w/PTSD and civilians	Diesel fuel, rotten egg, n-butanol	Varied	Not reported	Not reported
Kunihiro et al. (2017)	18, 19–24 years	Pocket NIRS Duo, DynaSense, Hamamatsu	2	PFC	Effect of Yomogi, lavender and Ylang ylang essential oil on brain activity	Yomogi, lavender, Ylang ylang	Varied	Varied	Not reported
Eto et al. (2014)	4, >65	FOIRE-3000, Shimadzu	46	PFC	Participants received different footcare treatments, footbath, a massage, a footbath with aroma oils, and an aroma oil massage. HbO2 measured	Lavandula angustifolia, tea tree, ravensara, palmarosa, and chamomile	Varied	Not reported	Varied
Bartocci et al. (2000)	23, mean 48 h ± 10.1	NIRO 300, Hamamatsu photonics	1	Left orbito-frontal gyrus	Assess olfactory cortex activity in awake infants receiving olfactory stimuli. Thirty second baseline, stimuli order: control, colostrum, vanilla	Control, colostrum, vanilla	**↑**	Not reported	Not reported
Saito-Iizumi et al. (2013)	7, mean 31.4 ± 4.5	ETG-4000, Hitachi medical	52	Frontal, temporal, parotid gland	Participants take part in two conditions, taste without odor, taste with odor, at different concentrations	Odor: Ethymaltol Taste: Sucrose dilutions 2–10%	**↑**	Not reported	Not reported
Schecklmann et al. (2011a)	27 ADHD, 22 control	ETG-4000, Hitachi medical	24	Inferior frontal & temporal	Olfactory sensitivity, discrimination & identification in ADHD with medication, ADHD without and controls, using Sniffin' Sticks	2-phenylethanol (rose)	**↑** ADHD with medication	Not reported	Not reported
Maharjan et al. (2019)	20, 25.05 ± 2.36	COVIDIEN INVOS OXIMETER, Model 5100C-PA	Not specified	OFC	Odor intensity tests (food odor) before and after low and high frequency nerve stimulation	Citrus, Banana, cheese, mushroom	Not specified	Not reported	Not reported
Ishimaru et al. (2004)	5, 32.69 ± 14.9	PSA500; Biomedical Science, Kanazawa	2	OFC	OFC activity observed during odor inhalation	b-phenyl ethyl alcohol, iso-valeric acid	**↑**	No change	Not reported
Bartocci et al. (2001)	20, 33.7 ± 2.3	NIRO 300, Hamamatsu photonics	2	OFC	15 infants exposed to disinfectant smell, 5 to detergent	Disinfectant, detergent	**↓**	No change	**↓**
Kobayashi et al. (2009)	13, (1) 28.9, (2) 46.9	Unspecified	22	Frontal lobe	Seated pasasive breathing through olfactometer for 5 s, followed by 55 s rest. Repeated 5 times	Isovaleric acid	**↑** Normosmic	No change	**↑** Normal
Kokan et al. (2011)	14, 19.6 years	Omega Monitor, BOM-L1W, OmegaWave Co	4	OFC	Passive inhalation through olfactometer, participant pushed button when odor detected	BPEA and citrus	Not reported	Not reported	Direction not stated
Schecklmann et al. (2011b)	29 (no age info)	ETG-4000, Hitachi medical	44	PFC	Olfactory function testing	2-phenylethanol, linalool	**↑** Controls & ADHD	Not reported	Not reported
Aoyama et al. (2010)	26, mean 5 days ± 1.8	NIRO-200, Hamamatsu photonics	2	OFC	fNIRS during sleep. Two groups, breast fed, bottle fed. All infants took part in both conditions. Odors presented *via* gauze detection	Maternal milk, bottle milk	**↑**	Not reported	Not reported
Hasegawa et al. (2013)	25, 27.0 ± 2.8	NIRO-200, Hamamatsu photonics	2	Frontal lobe	Participants chewed 3 gum types with different taste/odor combinations	Gum with and without lemon odor	**↑**		Not reported
Chen et al. (2021)	28 (no age info)	In-house built	2	PFC	Passive inhalation of odor	Lavender	**↑**	**↓**	Not reported
Maharjan et al. (2018)	18, 24.55 ± 3.8	COVIDIEN INVOS OXIMETER, Model 5100C-PA	2	Frontal	Odor Threshold Test (OTT) and Supra-threshold test (STT) with fNIRS	Snif test kit	No change	Not reported	Not reported
Ho et al. (2021)	12 (no age info)	ETG-4000, Hitachi medical	52	Frontal, temporal	Sniffin sticks 12 identification test	Apple, garlic, chocolate, sesame oil	No change	Not reported	Not reported
Fujii et al. (2007)	20, (1) 30–65 years (2) 24–48 years	Hitachi ETG-100	20	PFC	Prefrontal activity of Koh-Do experts whilst discriminating incense	Wood chips	Not reported	Not reported	Changes in expert group only
Kaimal et al. (2021)	17–54, M = 27.5 year	fNIRS Imager 2000S	16	PFC	VR with and without fragrance. Twenty hours sessions 1 week apart. Fragrance or on frag	Lavender, orange, juniper berry, patchouli	**↑**	Not reported	Not reported
Harada et al. (2006)	13, 23–31 years	NIRO 300 Hamamatsu Photonics	2	Frontal, temporal, parietal, occipital	Olfactory stimulation during odor exposure to pleasant and unpleasant stimuli	Vanilla, strawberry, scatol, distilled water	**↑**	Not reported	Not reported
Kim et al. (2022)	•CN: 55, 74.0 •MCI: 26, 74.0 •AD: 16, 76.5	Unspecified	7	Orbito-frontal cortex	Cognitive impairment detection paradigm. CN (cognitively normal), MCI (mild cognitive impairment), AD (Alzheimer's disease)	Unscented, peppermint	**↑** Controls	Not reported	Not reported
Tomono and Tomono (2020)	22, 21–24 years	NIRO-200 Hamamatsu photonics	2	Temporal	Passive inhalation of odors whilst watching video clips of others eating food	Bergamot orange, vanilla	**↑**	Not reported	Not reported
Mitsukura et al. (2021)	17 (no age info)	FOIRE-3000, SHIMADZU Co.	44	Frontal, occipital	fNIRS baseline recording and typing task before and after olive oil consumption	Olive oil, bread combo	**↓**	Not reported	Not reported
Jo et al. (2013)	26, 24 ± 1.8 years	OMM-2001, Shimadzu, Finometer device	47	Right hemisphere	Odor exposure followed by POMS questionnaire	Japanese plum blossom, control air	**↑**	Not reported	Not reported
Onuma et al. (2018)	12, mean 20	ETG-100, Hitachi medical	24	Frontal	Participants exposed to 4 ambient odors over 4 days. Each day faces evaluated based on femininity	3 floral odors, no odor	**↑**	Not reported	Not reported
Fladby et al. (2004)	29, 56–79 years	NIRO 300, Hamamatsu Photonics	2	Temporal	Inhalation of vanilla or sham water, activation measured in termporal region	Vanilla, water	Uncertain	Uncertain	Not reported
Igarashi et al. (2014)	20, 22.5 ± 1.6 years	TRS-20 Hamamatsu photonics	Not specified	PFC	3 odor stimuli presented for 90 s	Control, rose, orange oil	**↓**	Not reported	Not reported
Ikei et al. (2015)	13, 21.5 ± 1.0 years	TRS-20 system, Hamamatsu photonics	Not specified	PFC	Presentation of odor for 90 s, questionnaire following inhalation	Hinoki cypress oil, air	**↓**	Not reported	Not reported
Moss et al. (2018)	•Rose: 40, 23 years •Water: 40, 23.3 years	Oxymon, Artinis medical systems	12	Frontal	Drink rose water or water, fNIRS recording for 20 min while watching video	Rose water, water	No change	Uncertain	No change

↑ Indicates an increase in parameter, ↓ a decrease.

**Figure 2:**
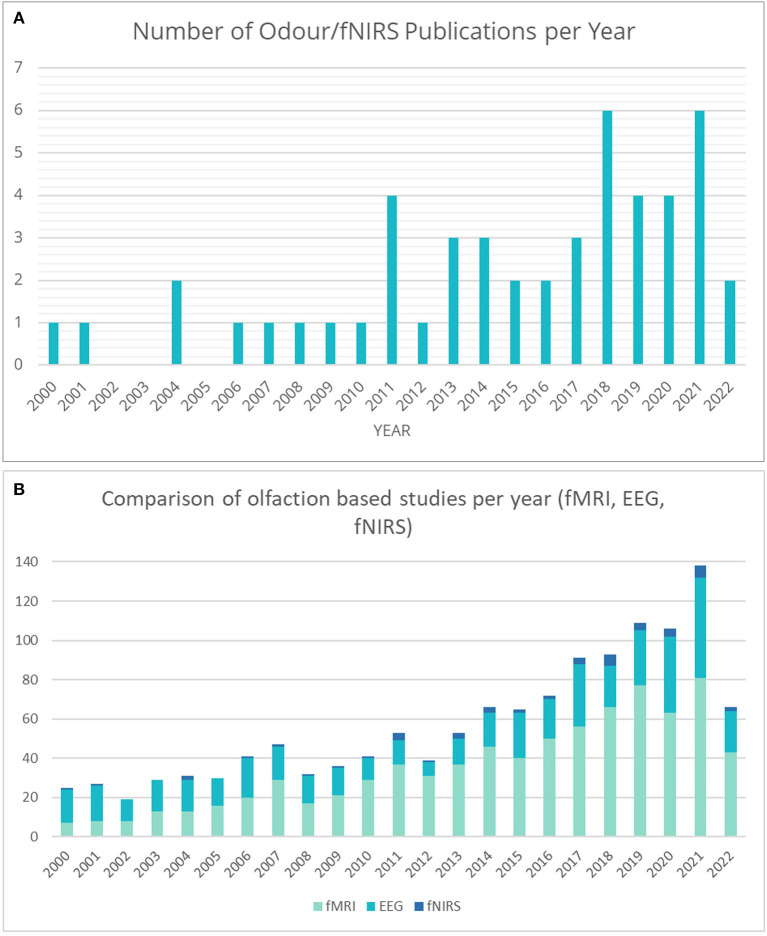
**(A)** Number of papers published by year involving fNIRS with odor stimuli. **(B)** A comparison of the number of papers published by year involving odor stimuli for fMRI, EEG, and fNIRS.

To our knowledge, this is the first systematic review collating odor and fNIRS studies. As can be seen in Figure 2A, the popularity of odor stimuli in fNIRS studies has shown a steady increase over the last 10 years, with spikes during 2018 and 2021. The drop in studies between 2019 and 2020 may potentially be attributed to the overall suspension of research during the COVID-19 crisis. In comparison to fMRI and EEG based odor related studies, fNIRS use is still in its infancy (Figure 2B), however, with the additional benefits fNIRS can bring, such as use in naturalistic settings, and the renewed interest in olfaction due to widespread anosmia experienced as a COVID-19 symptom, it is expected that odor research with fNIRS will continue to increase in popularity.

A total of 49 studies were included in this review, a summary of which, including details about instrumentation and stimulus, can be found in Table 1. With regards to the stimuli and technology used, all studies differed greatly. Overall, there was a preference for passive inhalation paradigms, with few studies implementing real-world designs. Of the real-world type studies found, two studies utilized the relaxing effects of essential oil aroma in a foot-care paradigm and one during a massage (Eto et al., 2014, 2015, 2016).

Five studies in total utilized a wearable device during testing, however, not all employed a real-world paradigm (Gramlich et al., 2017; Kunihiro et al., 2017; Rajagoplan, 2018; Tomono et al., 2021; Yamashita et al., 2022). The potential of wearable fNIRS devices in in-context scenarios will be explored in greater depth in the discussion sub-chapter.

**Figure 3:**
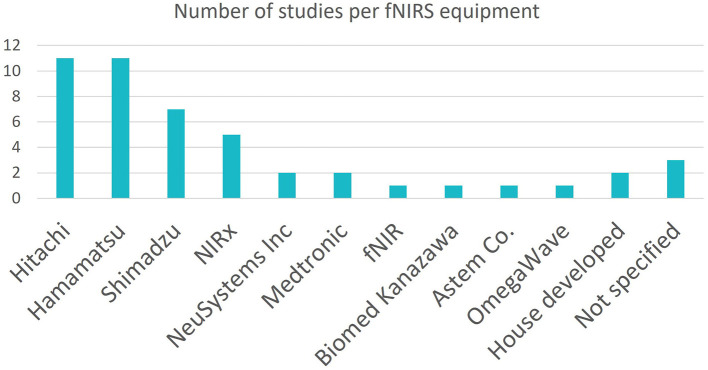
Indicating the numbers of studies which used each brand of device.

As seen in Figure 3, many different fNIRS instruments were utilized by the studies in this search, however the dominant manufacturers were Hitachi Medical and Hamamatsu Photonics, with the most popular device being the Hitachi Medical ETG-4000 fNIRS. This device allows for high density optode coverage. Its maximum channel number, 52, was utilized by three studies.

**Figure 4:**
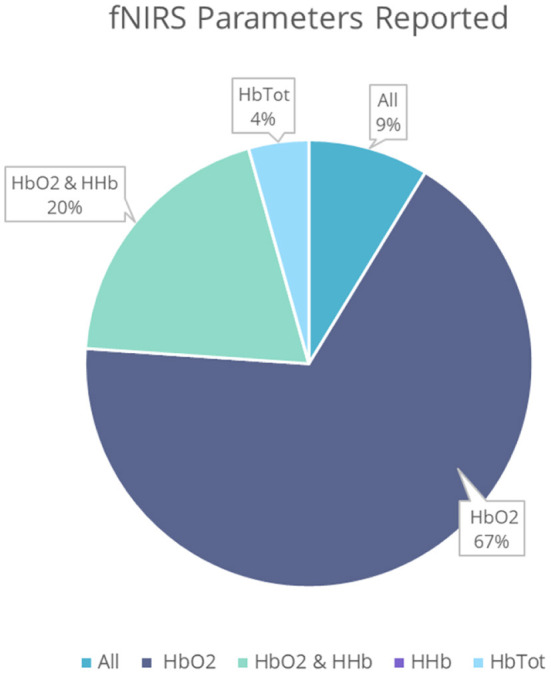
Percentage of papers reporting each fNIRS parameter.

With regards to the fNIRS parameters Δ[HbO2], Δ[HHb], and Δ[HbT], five studies reported findings for all three parameters, nine recorded Δ[HbO2] and Δ[HHb], 33 recorded Δ[HbO2] only and two recorded Δ[HbT] only. No studies observed Δ[HHb] only (Figure 4). Some papers depicted concentration changes in graphs rather that explicitly stating findings. Further analysis was predominantly conducted on Δ[HbO2] results. Figure 4 shows the percentage of studies reporting each hemodynamic parameter. For full parameter reporting, including use in each study and direction of activation, see Table 1. When comparing an odor stimulus to control, Δ[HbO2] was shown to generally increase with odor stimulus presentation, while, when reported, Δ[HHb] decreased.

**Figure 5:**
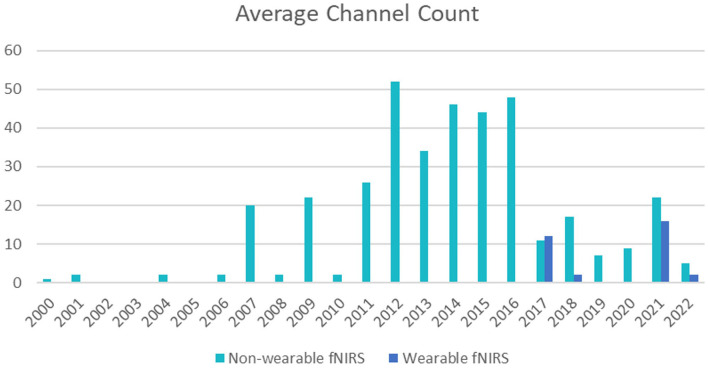
Indicates the average number of channels used per year between 2000 and 2022, excluding studies that did not provide channel number information. Wearable system channel numbers are shown in orange, and indicated recent favor toward wearable systems from 2017 onwards.

Channel numbers have generally increased since 2000, however, a notable drop in channel density can be seen from 2017 onwards (Figure 5). This reduction may coincide with the increased development and use of smaller, simpler, wearable systems in odor research, or use of small non-wearable systems such as the Hamamatsu Photonics TRS-20 system and NIRO-200, predominantly used in infant studies with 1-2 channels (Song et al., 2019; Tomono and Tomono, 2020; Muelbert et al., 2021). This is corroborated by the use of wearable devices in five studies, all conducted between 2017 and 2022. Devices used included the NIRSport NIRx device (Gramlich et al., 2017), the Pocket NIRS Duo, DynaSense, by Hamamatsu (Kunihiro et al., 2017), the Hb133 by Astem Co. (Yamashita et al., 2022), and the HOT-1000, WOT-100 by Neu Systems Inc (Rajagoplan, 2018; Tomono et al., 2021). The recent development of in-house custom systems for odor research has also shown a tendency toward low channel density, as in the case of Moein et al. (2020) and Chen et al. (2021). The need to combine fNIRS with other wearable systems, particularly virtual reality headsets, may also contribute to lower channel numbers (Kaimal et al., 2021). It should be noted that six studies did not report channel density information, so were excluded from Figure 5.

**Figure 6:**
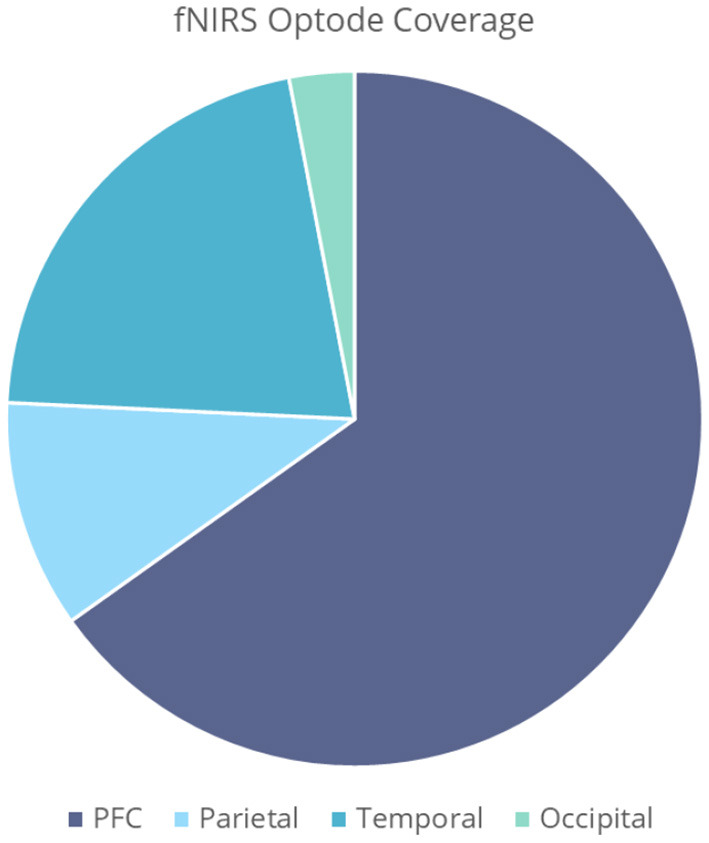
Optode coverage of PFC, temporal, parietal and occipital areas in 49 studies found.

**Figure 7:**
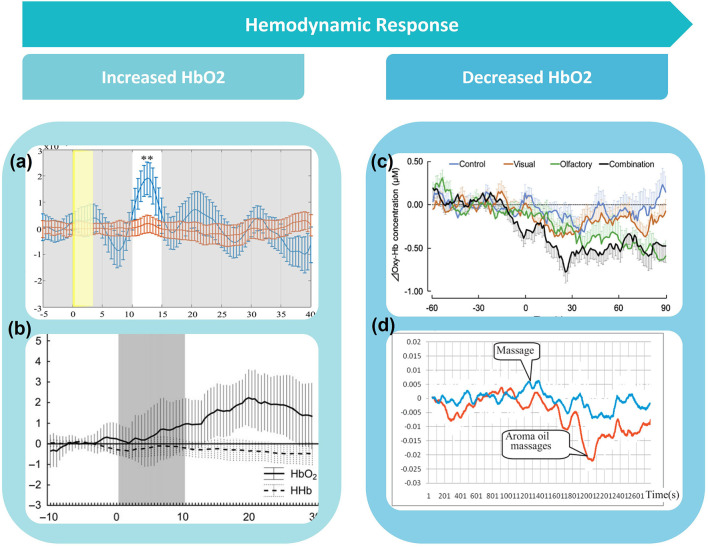
**(a)** Shows an example of a typical increase in HbO2 concentration (blue line) in response to odour, **(b)** a typical infant response to odour, **(c)** and **(d)** show a typical decrease in HbO2 in response to odour. Figures adapted from: **(a)** Harada et al. (2006), **(b)** Frie et al. (2017), **(c)** Song et al. (2019), and **(d)** Eto et al. (2014).

In general, there is high preference for prefrontal cortex (PFC) coverage (Figure 6). The close associations to regions involved in odor processing, as well as easy access for optode placement, grants the PFC high suitability for odor imaging. Other areas of interest include the primary and secondary olfactory cortices, found within the temporal regions of the brain, as well as the somatosensory cortex, found within the parietal lobe. The somatosensory cortex, found at Brodmann's areas 1, 2, 3a, and 3b, is responsible for processing sensory information, and has roles within odor processing and integration. A breakdown of regions of interest can be seen in Figure 6, with emphasis on the PFC/frontal regions, parietal, temporal, and occipital areas. Only four studies opted to register optode locations using digitization methods such as the FASTRAK Polhemus and Nirtrack by Shimadzu Co (Takakura et al., 2011; Hucke et al., 2018; Onuma et al., 2018; Xu et al., 2020). Additionally, one study presented MNI coordinates and AAL labels for all channel locations (Hucke et al., 2018). Three studies show interest in the parotid gland, also known as the salivary gland, found toward the temporal region of the brain. This region is often activated in the presence of food related odor stimuli. In this review, 3 studies utilize odor to enhance taste, e.g., ethylmaltol odor, soy sauce, and bonito flake aroma (Saito-Iizumi et al., 2013; Onuma et al., 2018; Tomono et al., 2021). The majority of studies were conducted on healthy, neurotypical adults. Some studies observed infants, as well as atypical populations with post-traumatic stress disorder (PTSD), attention deficit hyperactivity disorder (ADHD) and autism (Schecklmann et al., 2011a,b; Gramlich et al., 2017; Xu et al., 2020). Figure 7b demonstrates a typical infant response to odor stimuli, in this case a combination of hospital based alien odors (Frie et al., 2017). Response to odor in healthy adult controls can be seen in Figures 7a,c,d, highlighting the different responses that can be induced depending on paradigm. Paradigms involved in Figures 7c,d involve the intent to increase relaxation, and show a gradual decrease in Δ[HbO2] over time, whilst (Figures 7a,b) shows a typical increase in Δ[HbO2] in response to odor onset.

For the purposes of this review, the following sections are separated by population type; infant, atypical and typical. Within each section, details pertaining to odors used, demographics, protocols and hemodynamic findings will be stated. If studies show similarities or differences in any aspect of the protocol or results, this will be highlighted.

### Infant studies

Due to the artifact resistance and portability exhibited by fNIRS, many papers focused on infant studies. In this literature search, six infant studies investigated odor with fNIRS. Most studies reported observing multiple infant age groups. Three studies investigated odor effects on preterm infants; pre-term, very pre-term (Frie et al., 2017, 2019; Muelbert et al., 2021). The remaining three studies observed full-term infants (Bartocci et al., 2000, 2001; Aoyama et al., 2010). Most papers observed activity during sleep (Aoyama et al., 2010; Frie et al., 2017, 2019), whilst three studies observed wakefulness (Bartocci et al., 2000, 2001; Muelbert et al., 2021). In terms of odors, four of the six studies used maternal scents such as breast odor, breast milk odor and maternal colostrum as the stimulus (Bartocci et al., 2000; Aoyama et al., 2010; Frie et al., 2019; Muelbert et al., 2021). These odors were either collected and delivered on a cotton pad, gauze or cotton bud. Other odor stimuli include unnatural aromas experienced in hospital situations, such as disinfectant, detergent and hand cleaner. These odors were found to be capable of eliciting trigeminal pain responses (Bartocci et al., 2001; Frie et al., 2017).

**Figure 8:**
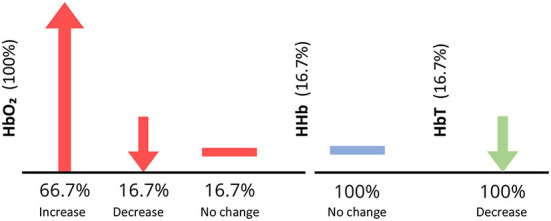
Vertical axis shows the percentage of infant studies that reported each parameter. Horizontal axis shows the percentage of papers within each parameter [HbO_2_, HHb, and HbTotal (HbT)] reporting an increase, decrease or no change in activity in response to an odor, for infant studies only. For example, all papers reported HbO_2_, however, only 66.7% observed an increase in HbO2, illustrated by an upwards arrow. Downwards arrows indicate a decrease in parameter concentration, and a horizontal line depicts no change in parameter concentration.

Two studies analyzed both Δ[HbO2] and Δ[HHb], three analyzed Δ[HbO2] only, and one analyzed all three parameters. Apart from Bartocci et al. (2001) and Muelbert et al. (2021), all studies found an increase in Δ[HbO2] in the OFC region, in response to odor stimuli. Muelbert et al. (2021) found no significant change in Δ[HbO2] after odor exposure, while Bartocci et al. (2001) demonstrated a decrease in Δ[HbO2] and Δ[HbT] in response to hospital odor stimuli. Activation also varied within stimulus types. Interestingly, one study using maternal odors, found significantly higher activation in response to maternal odor than a control (Aoyama et al., 2010), with the other finding either a slight increase in Δ[HbO2] or no change at all (Bartocci et al., 2000). Findings are summarized in Figure 8.

**Figure 9:**
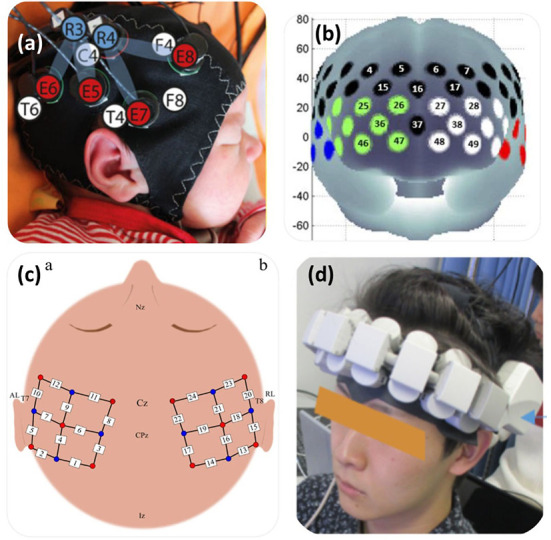
Depicts the fNIRS optode arrangements for 4 studies. **(a)** configuration used to image full-term and pre-term infants in response to maternal odours, targeting the primary olfactory cortex, found between the orbitofrontal cortex and piriform cortex. **(b)** in the study of individuals with covid-19 induced olfactory impairments, PFC and temporal coverage was utilised (Ho et al., 2021). **(c)** in a study of healthy participants, bilateral coverage was implemented (Hucke et al., 2018). **(d)** a small 16-channel wearable system observed PFC activity in response to food odours whilst watching food related videos Tomono et al. (2021). Figures adapted from: **(a)** Frie et al. (2017), **(b)** Ho et al. (2021), **(c)** Hucke et al. (2018), **(d)** Tomono et al. (2021).

This contradiction may be due to age differences of the experimental population, with Aoyama et al. (2010) opting to use infants with a mean age of 5 days ± 1.8, compared to 48 h ± 10.1 in Bartocci et al. (2000). fNIRS optode coverage ranged from 1 to 8 channel configurations. Only one paper adopted a single channel device (Bartocci et al., 2000). Both Bartocci et al. (2001) and Muelbert et al. (2021) utilized Hamamatsu Photonics dual channel oxygenation monitors. An example of an infant fNIRS configuration can be seen in Figure 9a, in the study by Frie et al. (2017).

### Atypical population studies

Eight studies investigated odor effects using atypical populations, the majority of which investigating features such as olfactory perception, discrimination and identification in populations with underdeveloped, altered or damaged olfactory processing systems. The atypical populations included those with autism spectrum disorders (ASD) (Xu et al., 2020), combat related PTSD (Gramlich et al., 2017), Alzheimer's Disease and mild cognitive impairment in the elderly (Kim et al., 2022), dysosmia (Kobayashi et al., 2009), multiple chemical sensitivity (MCI) (Azuma et al., 2016), and ADHD in children and adults (Schecklmann et al., 2011a,b). There has also been interest olfactory changes due to COVID-19 contraction (Ho et al., 2021). Methods of odor delivery varied between studies, with 50% of papers opting for passive inhalation such as “Sniffin' Sticks,” and the remaining using pulse ejection systems or olfactometers.

All eight odor studies reported Δ[HbO2]. Five studies observed increased activity in the presence of an odor, however, this activity was significantly lower than activity observed in control participants. Using individuals with MCI, Azuma et al. (2016) reported a significantly higher response to low intensity household products when compared to control participants, indicating heightened perception. Three studies also reported Δ[HHb].

**Figure 10:**
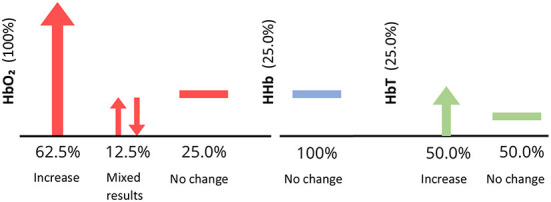
Figure depicts number of papers reporting Δ[HbO2], Δ[HbT], and Δ[HHb] (vertical axis) for atypical studies only. The percentage of papers reporting each response (horizontal axes) is denoted by arrows, e.g., upwards indicates increase in concentration. A number of studies included here did observe increased activity in response to odor in atypical populations, however, this response was significantly diminished compared to controls.

Only one paper opted to report all three parameters, however, did not find any significant differences in activity in response to odor in any parameter (Kobayashi et al., 2009). Figure 10 shows a breakdown of parameter results. The fNIRS optode coverage for these odor studies ranged from 7 to 52 channel configurations. Figure 9b demonstrates an optode configuration for a study using persons on the autism spectrum (Xu et al., 2020). Optode coverage mostly inspected the PFC, OFC, and some in temporal regions.

No papers mentioned the use of short-channels or extra systemic measurements. One study utilized the mobile NIRx device, NIRScout-88 (Gramlich et al., 2017). Behavioral data was collected from various tasks and questionnaires, such as the Somato-Sensory Amplification Scale (Azuma et al., 2016), Wechsler Intelligence Scale for Children, Wechsler Adult Intelligence Scale, sensory profiles, olfactory measurement and Childhood Autism Rating Scale (Xu et al., 2020), odor and audio hedonic and intensity ratings (Gramlich et al., 2017), impulsivity, depression and intelligence scales (Schecklmann et al., 2011a), and visual analog scales for valence and intensity of odor (Schecklmann et al., 2011b).

### Healthy population studies

**Figure 11:**
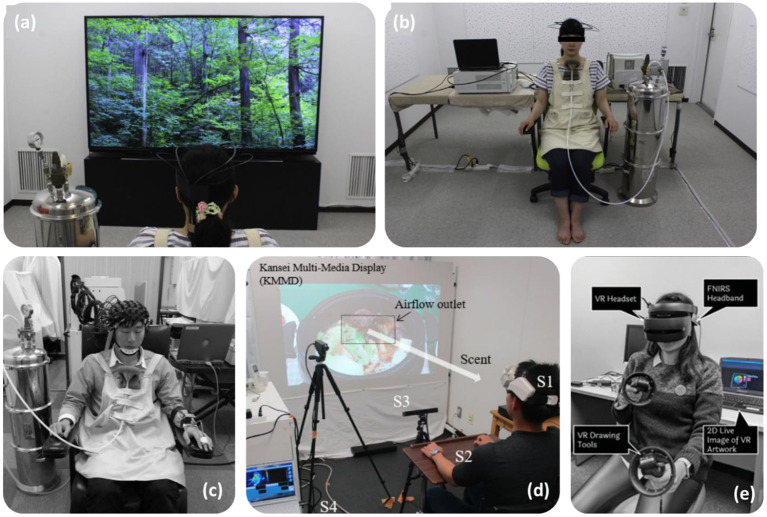
Depicts fNIRS task set-ups for four studies, two include viewing videos, one using virtual reality. Figures adapted from: **(a,b)** Song et al. (2019), **(c)** Jo et al. (2013), **(d)** Tomono et al. (2021), and **(e)** Kaimal et al. (2021).

Studies involving healthy adult populations and odorant stimuli account for 35 studies of the 49 found, one of which utilizes EEG with fNIRS (Invitto et al., 2019). Odor delivery methods varied greatly between studies. Three studies opted for olfactometer odor delivery (Kokan et al., 2011; Hucke et al., 2018; Invitto et al., 2019). Most studies opted for odor containers, such as bottles (Saito-Iizumi et al., 2013; Maharjan et al., 2019; Moein et al., 2020; Chen et al., 2021; Yamashita et al., 2022), test tubes (Fladby et al., 2004), odor filled bags (Igarashi et al., 2014; Ikei et al., 2015), climate controlled room (Figures 11a,b) (Song et al., 2019) and pressure containers (Figure 11c) (Jo et al., 2013). Jo et al. (2013) incorporated naturally found stimuli, e.g. fresh flower petals, collected on the day of testing as a stimulus, allowing the odor to diffuse into the air within a pressure container. Five studies implemented odor delivery mechanisms aiming to diffuse odor into the environment around the participant, including below the participants nose with the use of a wearable odor tray (Onuma and Sakai, 2018; Song et al., 2019; Tomono and Tomono, 2020; Kaimal et al., 2021; Tomono et al., 2021). One of these studies, Tomono and Tomono (2020) expelled odor vapor via a airflow generator toward the participant, whilst also measuring odor concentration produced to check odor concentration consistency (Figure 11d). Unlike the atypical research presented previously, the odor delivery mechanism, Sniffin Sticks, was not used in studies involving healthy adult populations. A similar instrument, known as the Snap and Sniff Olfactory Test System, utilizes pen-like odor delivery devices, and was used in one study investigating olfactory improvement (Maharjan et al., 2018). Three studies utilized simple smelling strips to investigate the effect of essential oils on brain activity, as well as other unpleasant and pleasant odors such as isovaleric acid and strawberry, respectively (Ishimaru et al., 2004; Harada et al., 2006; Kunihiro et al., 2017). Two studies opted to use odors on material substances such as t-shirts and odor pads (Nishitani et al., 2014; Onuma et al., 2018). The cotton pads used in Onuma et al. (2018) were soaked in soy-sauce odor and placed out-of-sight within cups containing taste stimuli, to investigate the influence of odor on taste perception. One study opted to use a fragrances liquid, rose water, as a stimuli (Moss et al., 2018). A significant proportion of studies incorporated odor in an in-context paradigm, e.g., foot massage using essential oils (Eto et al., 2014, 2015, 2016), presentation with or in food or drink (Matsumoto et al., 2012; Onuma et al., 2018; Rajagoplan, 2018; Mitsukura et al., 2021), chewing gum (Hasegawa et al., 2013), incense (Fujii et al., 2007), and household spray (Tanida et al., 2008). One study aimed to develop a novel odor delivery system. ReminiScentia, developed by Casillas-Figueroa et al. (2020) dispenses an odor selected by the participant during the task, by using the digital interface. The device was used to run odor based paradigms, such as scent-guided navigation, by presenting odor from different directions to influence task choices. Only one study did not directly involve odor exposure, but instead induced the perception of odor by other means. Takakura et al. (2011) induced the presence of garlic odor on participants *via* the injection of intravenous thiamine propyl-disulphide, which is known to induce this effect unintentionally. Some studies utilized tasks, e.g., perception, face rating, within the odor paradigm (Onuma and Sakai, 2018; Onuma et al., 2018; Rajagoplan, 2018; Invitto et al., 2019; Moein et al., 2020). Another study investigated the effect of crossmodal haptic and olfactory stimulation combination with a paradigm involving 3D models (Invitto et al., 2019). Yamashita et al. (2022) also utilized a combinatory study, with visual light displays as well as odor. Mitsukura et al. (2021) employed a cognitive typing task prior to and after olive oil consumption. Similarly, Tanida et al. (2008) tested mental arithmetic prior to and after exposing participants to a floral fragrance for 4weeks. Virtual reality was implemented in one study with and without a relaxant fragrance to the influence of odor on creativity and the ability to free-draw (Kaimal et al., 2021). This study can be seen in Figure 11e.

**Figure 12:**
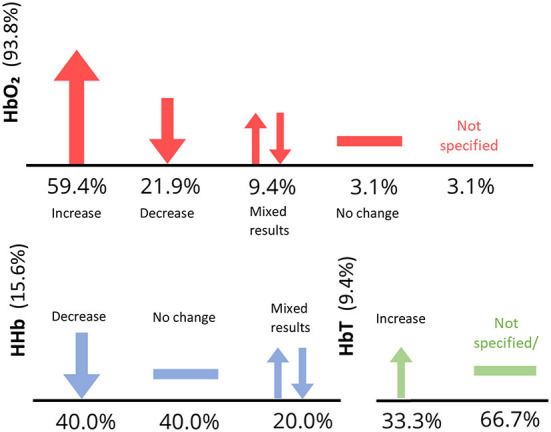
Figure depicts number of papers reporting Δ[HbO2], Δ[HbT], and Δ[HHb] (vertical axis) for typical studies only. Note that some studies did not report findings.

Four studies analyzed both Δ[HbO2] and Δ[HHb] with varying results. One study found decreased activity in response to odor (Moein et al., 2020), increased Δ[HbO2] and decreased Δ[HHb] (Fladby et al., 2004; Saito-Iizumi et al., 2013) and increased Δ[HbO2] with no change in Δ[HHb] (Invitto et al., 2019). Three other studies reported findings for all three parameters, finding increased Δ[HbO2], Δ[HbT], and decreased Δ[HHb] (Hasegawa et al., 2013; Eto et al., 2014) and no significant effect of fragrance for all parameters (Moss et al., 2018). The vast majority of studies reported and analyzed Δ[HbO2] only. Eleven studies observed increased Δ[HbO2] in response to odor stimulus, however this was odor dependent. For example, Ylang ylang odor increased Δ[HbO2], however, within the same study, Lavender odor decreased Δ[HbO2] (Kunihiro et al., 2017). Δ[HbO2] change was found to also be dependent.

Nishitani et al. (2014) observed increased Δ[HbO2] in mothers in response to infant odor, a finding which was not replicated in non-mothers. Seven studies observed significant decreases in Δ[HbO2] in response to various odors, e.g., relaxing essential oil odors during a massage (Eto et al., 2015, 2016), olive oil odor (Mitsukura et al., 2021), forest odor (Song et al., 2019), Hinoki oil (Ikei et al., 2015), rose and orange oil (Igarashi et al., 2014), and floral spray (Tanida et al., 2008). It is important to note that some studies observed no significant effect of odor on cerebral hemodynamics (Maharjan et al., 2018; Onuma and Sakai, 2018; Kaimal et al., 2021; Tomono et al., 2021). Two studies reported Δ[HbT] only (Fujii et al., 2007; Kokan et al., 2011). A summary of all parameter results can be seen in Figure 11.

Only three studies mentioned the use of multi-distance optode configurations in the experimental paradigm, however, details regarding the short-channels were unclear (Kokan et al., 2011; Maharjan et al., 2019; Song et al., 2019). All studies but one (Bartocci et al., 2000) incorporated at least two channels within the multi-channel configurations. Two examples of fNIRS device used in healthy participant studies can be seen in Figures 9c and d, one of which being a 16-channel wearable system (Tomono et al., 2021). In total, four of the healthy population studies utilized wearable devices, e.g., HOT-1000 by Neu Systems Inc for a study involving consumption of green tea (Rajagoplan, 2018), Pocket NIRS Duo, DynaSense, Hamamatsu to investigate essential oils (Kunihiro et al., 2017), WOT-100 NeU Corporation for investigating odor effect whilst watching food related videos (Tomono et al., 2021) and fNIRS Hb133, Astem Co., Ltd. to observe effects of odor and light on activity (Yamashita et al., 2022). Not all studies utilized physiological monitoring along with fNIRS. Only eight studies included systemic measurements in their investigations, including heart rate and heart rate variability (Tanida et al., 2008; Jo et al., 2013; Igarashi et al., 2014; Ikei et al., 2015; Song et al., 2019; Tomono et al., 2021), respiration rate, blood pressure (Jo et al., 2013), facial skin temperature (Tanida et al., 2008), saliva flow (Tomono and Tomono, 2020; Tomono et al., 2021), and muscle movement *via* electromyogram (Hasegawa et al., 2013). Most studies included some element of behavioral data collection. Two used semantic differential methods to determine thoughts on the odor stimuli presented (Jo et al., 2013; Song et al., 2019). Some studies included questionnaires relating to perceptions of odors, utilizing visual analog scales and ratings of saltiness, sweetness, comfort and relaxation, as well as the Profile of Mood States questionnaire. The Standard Trait Anxiety Index to measure transient stress levels was used in Tanida et al. (2008) to measure stress during a cognitive task, before and after a 4 week period of fragrance exposure.

## Discussion

### Interpreting studies involving fNIRS and olfaction

To our knowledge, this is the first systematic review collating odor based fNIRS research. The results of this search produced 49 papers, information from which has been extracted and discussed above. This review briefly introduces fNIRS experimental paradigms used for studies involving odor, for example, the odor detection, identification and evaluation methodologies used in atypical studies, effect of odor on cortical activity in passive paradigms, as well as during cognitive performance tasks. Despite the varied experimental paradigms, population groups and stimuli, overall there appears to be some agreement between studies. For example, odor studies used different odor delivery methods, however, most opted for passive inhalation paradigms, e.g., diffusion, within containers such as bottles and test tubes, smelling strips, and scented cotton pads. Only a few studies opted to use olfactometers, perhaps due to ecological invalidity. Generally, in the presence of odor, a significant effect on activity can be found, however the direction of this effect varies depending on factors such as participant type, odor family and concentration. The use of multichannel configurations in almost all studies can confirm that the presentation of an odor elicits a change in activity, most often an increase in HbO2 concentration, in specific regions such as the orbitofrontal cortex. This is in line with the understanding that the orbital cortex receives direct axonal connections from the primary olfactory cortex (Chen et al., 2021).

### Population differences and size

Differences between population types can also be identified using fNIRS and odor. In cases of atypical populations, diminished activity was seen in atypical populations in response to odor compared to controls, suggesting altered odor processing in atypical populations. In the case of Kim et al. (2022), the possibility of using fNIRS and odor as a combinatory diagnostic tool was explored, based on the understanding that discrimination between odors is significantly diminished in persons with cognitive impairment or Alzheimer's disease. The method was found to be an effective measure of decline in suspected impaired participants, as odor induced oxygenation and discrimination ability was significantly reduced as the severity cognitive impairment increased, as measured by a battery of cognitive function tests. Similarly to other fNIRS studies involving infants, the results have found differences in response to odors between infants of different ages, even in the case of the same odor type being used, highlighting the importance of considering participant age carefully when recruiting from infant populations. No significant differences between ages of adult populations were reported in the papers.

The odor differences between males and females has been investigated in recent years, finding increased sensitivity to smells in females than males (Sorokowski et al., 2019). Within this review, differences have also been found in cortical activity between males and females. Multiple studies observed significant interactions of fragrance with gender, predominantly indicating lower activity in response to an odor in females than in males, in both adults and infants (Eto et al., 2016; Frie et al., 2019; Kaimal et al., 2021; Muelbert et al., 2021). Interestingly, differences within a gender were also found when exposed to certain odor stimuli. Nishitani et al. (2014) observed significantly higher activity in women who were mothers, than women who were not, in response to infant odor on t-shirts. These subtle, but significant differences between participants should be kept in mind during the recruitment process and experiment design phase.

**Figure 13:**
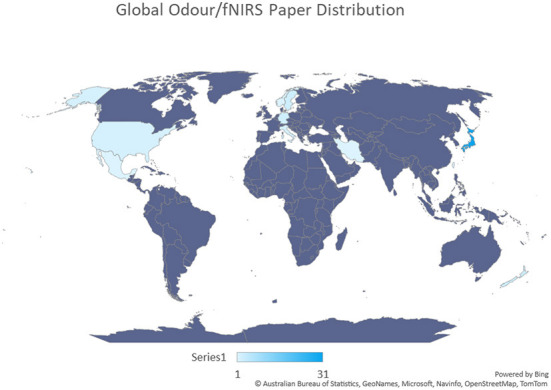
A graphical representation of country contributions toward odor based fNIRS studies, generated from the data in Table 1. The first author and affiliation for each study was used to create this graph. Darker blue shades indicate a higher number of papers generated from a region. Purple shades indicate no fNIRS odor papers. From this figure it can be seen that a significant portion of fNIRS odor studies are conducted in Japan.

One significant limitation of the majority of odor based studies is the population size used. Interestingly, infant and atypical population studies involved generally larger population sizes compared to research using healthy populations, with an average population size of 32 and 37 participants per study, respectively. On average, healthy studies only used 17 participants per study. To increase generalizability and reliability, studies should consider incorporating significantly larger sample sizes. Due to the highly personal and emotional nature of odor, larger sample sizes will also reduce the effect of individual differences in age, gender, past experiences and odor hedonics on subsequent results. Odor hedonic perception is an umbrella term encompassing the pleasant or unpleasant character of an odor, known as hedonic valence, and liking toward said odor. Collecting such information during recruitment or experiment can allow variations between groups with different hedonic characteristics to be determined. Additionally, during the recruitment period, screening participants for olfactory dysfunction should be considered, as underlying or unknown olfactory deficiencies or deviations from normal function have the potential to skew results. The majority of atypical studies in this review have used various tests to do this, whilst most studies involving healthy populations have not done so. Multiple olfactory function techniques have been discussed in this review, including the methodology implemented by Azuma et al. (2016), wherein odor detection and recognition were assessed using an olfactometer for individuals with heightened olfactory sensitivity. Presented at increasing concentrations, the detection threshold is determined as the point at which the participant notices the scent, but does not recognize. Identification of the odor is labeled as the recognition threshold. The averaging of information from this test gives researchers an indication of olfactory acuity for each participant. Other testing measures include the University of Pennsylvania Smell Identification Test (UPSIT), a commercially available olfactory function test. This test comes in the form of a booklet, with scratchable strips containing odor alongside related multiple choice questions. The UPSIT is commonly used in studies involving Parkinson's disease and Alzheimer's, however, in this review, it was used to test for and recruit Normosmic participants (Moein et al., 2020). It is important to note that there are various versions of this test available for use with different geographic demographics, including versions for Britain, France, Italy, Korea, and China. One potential drawback of the current cohort of research in fNIRS and odor is the large portion of participants belonging to the same or similar participant demographics in East Asia (Figure 13). Currently, no research was found investigating odor in South Asian regions, despite the known long history with odor in relaxation practices. Additionally, no research has been using participants from Africa or South America, both vast and culturally diverse regions, with different odor palettes depending on environment, lifestyle and food.

### Odor types

Another contributor toward the varied results produced is the varying responses elicited between odors, causing the interpretation of cerebral responses of odors in fNIRS studies to be complicated. As mentioned previously, odors can belong to different odor classes and families. Each family can evoke unique responses in both brain and behavior. Some fragrances are able to increase activity and invigorate mental state, such as peppermint or rosemary, whereas others have more sedative and calming effects like lavender and chamomile. The odor chosen for each study can therefore dramatically impact the hemodynamics measured. Additionally, individual preference toward each odor may also contribute to the diverse Δ[HbO2], Δ[HHb], and Δ[HbT] results shown in this search. This should be taken into consideration when interpreting fNIRS data from these studies. A few papers did address the differences between odors rather than vs. control substances, however, currently there does not seem to be enough odor based fNIRS studies to be able to group papers by odor family for wider interpretation. Additionally, no mention was made by any papers regarding specific odor families used, any underlying known effects, or the specific reasons why odors were selected. This may indicate a limited understanding of odors or fragrances selected. More research involving specific odor families would be beneficial in this field, not only for industry and marketing benefits, but also for the collective improvement of general wellbeing.

Essential oils have been found to have significant effects on brain activity, as well as behavior and mood, however, only a few studies utilized essential oils with fNIRS. Three studies utilized a blend of Lavendula angustifolia, tea tree, Ravensara, Palmarosa, and chamomile essential oils (Eto et al., 2014, 2015, 2016). Three studies utilized the essential oils of native trees and plants in the regions, such as in Japan with the use of cypress tree and Yomogi essential oil in studies relating to the effect of Cypress oil on forest related visual stimuli (Ikei et al., 2015; Song et al., 2019) the interaction between colored light and odor (Yamashita et al., 2022) and the stress relieving effects of Yomogi oil (Kunihiro et al., 2017). One further study utilized a blend of essential oils; lavender, orange, juniper berry, patchouli and Ylang ylang (Kaimal et al., 2021). This particular study investigated the effect of the various fragrances mentioned on rote tracing and creative self-expression using a virtual reality device. Although no significant effect of fragrance was found in this case, differences in gender and age of the 16 participants in response to fragrance were noted. This indicates that more research is needed using larger population sizes to determine the hemodynamic effects of essential oils and aromatherapy with fNIRS. Wearable fNIRS systems should be utilized to a greater extent within such studies to alleviate restriction further, broadening the scope of odor related contextual research.

### Study design

The most common fNIRS odor paradigm involved passive inhalation whilst in a seated and comfortable position (Bartocci et al., 2000, 2001; Fladby et al., 2004; Ishimaru et al., 2004; Harada et al., 2006; Kobayashi et al., 2009; Aoyama et al., 2010; Frie et al., 2017, 2019; Hucke et al., 2018; Casillas-Figueroa et al., 2020; Ho et al., 2021). Some studies also utilized questionnaires during or after the fNIRS recording phase to measure odor stimuli hedonic value and intensity for each odor presented (Kokan et al., 2011; Jo et al., 2013; Nishitani et al., 2014; Azuma et al., 2016; Gramlich et al., 2017; Kunihiro et al., 2017; Chen et al., 2021; Tomono et al., 2021). A few studies implemented tasks requiring the participant to identify the moment of odor detection and recognition during testing, either verbally, or using a button (Kokan et al., 2011; Moein et al., 2020; Xu et al., 2020). Other studies used olfactory function tests as the basis for odor stimuli implementation (Schecklmann et al., 2011a,b; Azuma et al., 2016; Kim et al., 2022). Multiple studies employed food or drink as a means to introduce odor stimuli (Matsumoto et al., 2012; Hasegawa et al., 2013; Onuma et al., 2018; Rajagoplan, 2018; Tomono and Tomono, 2020; Mitsukura et al., 2021; Muelbert et al., 2021; Tomono et al., 2021).

Overall, most studies utilized block design paradigms to implement odor stimuli. Odor stimuli presentation durations varied greatly between studies, however, most opted to add sufficient lengths of rest between odor stimuli to reduce carry-over effects. Rest periods were most often between 20 and 40 s long, with odor stimuli periods most commonly between 10 and 30 s. Odor stimuli repeats and block designs were not always included, particularly in cases where odors were diffused into the testing environment over time rather than delivered on various materials (Onuma and Sakai, 2018; Song et al., 2019; Kaimal et al., 2021) or in infant studies. One study of import used a longitudinal design, testing cognitive performance before and after a 4 week fragrance intervention using a floral spray (Tanida et al., 2008). One study utilized a climate controlled cubicle, displaying images of Hinoki cypress trees with and without Hinoki essential oil (Song et al., 2019). Although this method did find significantly reduced HbO2 in the trial with Hinoki oil, it would be interesting to replicate some aspects of this study within a natural Hinoki tree environment during a peaceful forest walking paradigm.

### Physiological measurements

Tanida et al. (2008) also utilized facial sebum secretion measures as an indicator of stress levels, finding decreased sebum production after 4 weeks of floral fragrance exposure compared to controls. Kunihiro et al. (2017) tested salivary α-amylase before and after inhalation of Yomogi essential oil. α-amylase is a strong indicator of stress levels, and in this case, was found to be lower in participants inhaling Yomogi oil than in controls, although not significant.

Some studies included additional measures of heart rate, breathing rate and oxygen saturation using external devices such as the Radical 7 (Masimo) and GE DASH-4000 monitor (GE Healthcare) (Frie et al., 2017) the Tsuyama MGF KK, photoelectrical sensor (Tanida et al., 2008), and pulse oximetry by Hewlett-Packard, Germany (Bartocci et al., 2001). Jo et al. (2013) measured heart rate, as well as blood pressure and heart rate variability using a finometer device and electrocardiogram, AC-301A; GMS Corp., Japan. Heart rate variability gives an indication of variations in heart rate rhythm. Any changes observed in heart rate can be due to changes in mental and emotional state.

Another measure utilized by one study in the review observed pupil dilation. Pupillary diameter can be used as a measure of participant mental state, able to reflect behaviors such as level of interest. Used in a paradigm involving food videos, the eye recorder EMR ACTUS by nac Image Technology Corporation was used by Tomono et al. (2021) to monitor participant pupil dilation and line-of-sight. Generally, individuals tend to look toward an object or person of particular interest, which can give an indication of a particular subjects preferences.

Interestingly, no studies utilized galvanic skin response (GSR) as a measure of relaxation or stress. The GSR signal is a direct measure of the action of sympathetic nerves on the sweat glands of the skin. An increase in arousal, due to fear, joy or stress causes the heightened activity of sweat glands. The increased production of sweat on the surface of the skin elevates electrical conductivity, which in turn can be detected GSR monitors. Due to the nature of arousal, the exact identity of the emotion experienced cannot be determined from galvanic skin response alone, only the presence and intensity of it.

The incorporation of extra physiological measures such as facial sebum secretion, cortisol testing, blood pressure and eye tracking, galvanic skin response, and dilation should be used in future studies aiming to investigate the influence of odor on mental state and behavior.

### Regions of interest

The olfactory bulb and deeper associated olfactory structures, such as the amygdala, are not within reach of fNIRS devices, however, other brain regions can be influenced by odor stimuli, and are penetrable by fNIRS. Studies in this review designed configurations to predominantly cover the prefrontal cortex (PFC), which is known to be structurally connected to the olfactory pathway (Yamaguchi, 2017). Many studies in this review observed significant changes in PFC activation in response to an odor.

Tanida et al. (2008) observed significantly decreased HbO2 after a 4 week fragrance intervention, indicating the connection between the PFC and the effect of fragrances on stress. The orbitofrontal cortex (OFC), temporal and parietal areas were also favored. The OFC is associated with odor perception and identification, whilst the temporal region, involved with some aspects of olfactory memory, is often imaged to capture olfactory cortex and parotid gland activity (Savage et al., 2002). The olfactory pathway is also closely linked to regulatory areas within the brain associated with emotion, memory, and learning. This interconnectivity allows for a multitude of paradigms and channel configurations to be applicable to fNIRS and odor studies.

### Channel configuration and multi-distance channels

The use of multichannel configurations provides more brain coverage and increases the ability to localize induced activity. Almost all studies in this review opted to use multichannel probe designs, with only one study using a one channel design (Bartocci et al., 2000).

In addition to multichannel configurations, studies may make use of multi-distance channel configurations. Using various source-detector distances allows the probing of various depths of the human head. At present, the optimal source-detector distance 30 mm. This distance allows the interrogation of the cortical areas of the brain. Shorter distance separations can be utilized to record scalp hemodynamics, which can interfere with measurements of cortical areas. Using short channels, scalp hemodynamics can be removed from the measured fNIRS data, with the intention of the remaining data being cortical activations only. This technique also improves the signal to noise ratio (SNR) of the data. This regression technique is becoming increasingly common in fNIRS research, however, only one study included in this literature review mentioned the use of an fNIRS device with multi-distance channel capabilities. Although short channels were mentioned, no information regarding their implementation or regression was provided in the data preprocessing or analysis segments of the paper (Maharjan et al., 2019). If optode space is available, future studies should aim to utilize short channels in the experimental design.

### Wearable fNIRS

Despite having limited penetration depth and spatial resolution when compared to fMRI, fNIRS has the advantage of artifact resistance and portability, creating immense scope for future odor based research. Using lightweight, wearable fNIRS devices, such as those used by Gramlich et al. (2017), Kunihiro et al. (2017), Rajagoplan (2018), Tomono et al. (2021), and Yamashita et al. (2022), experimental paradigms can be designed to take place in the context of particular smells, e.g., in a forest, car or a perfume store, heightening applicability of subsequent findings, and allowing participant to have more natural and unregulated responses to stimuli. To support a more event-related paradigm, analysis methods such as AIDE, Automatic IDentification of functional Events, have been developed, and can be used in the place of conventional analysis (Pinti et al., 2017).

### Pre-processing and analysis

As with the experimental paradigms and findings described previously, preprocessing and analysis methods also differ greatly between papers. Note that all studies pre-processed fNIRS data, but not all reported the pre-processing steps applied. Where reported, fNIRS data was filtered to remove systemic and equipment interferences, such as heart rate and breathing rate. The low-pass and high-pass filter values varied greatly between studies. To remove signal drift and slow oscillations, the studies reported here use low pass, high pass, and band pass filters to remove both high and low frequency noise and artifacts. The parameters used for these filters are dependent on study design, therefore block length should be considered before deciding this. One papers used a Finite Impulse Response filter (Kaimal et al., 2021). For signal smoothing, multiple studies implemented a moving average smoothing technique which removed short term artifacts from the data (Schecklmann et al., 2011a,b; Eto et al., 2014; Frie et al., 2017, 2019; Ho et al., 2021). Other artifact identification, removal or correction techniques include using the MARA correction algorithm, Correlation based signal improvement (CBSI), visual inspection and epoch removal, removal with Principle Components Analysis, correction with linear interpolation, and spline interpolation.

**Figure 14:**
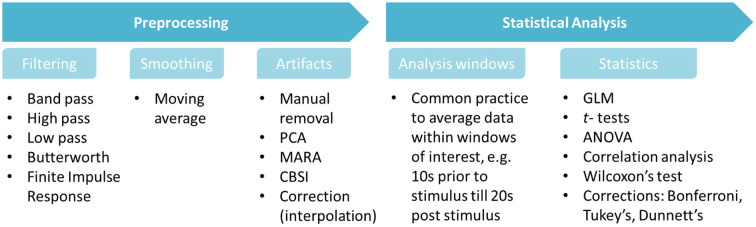
Summarized preprocessing and analysis pipeline for odor based fNIRS data, generated from papers in Table 1. It depicts commonly used functions and processes when handling fNIRS data.

Prior to statistical analysis, it is often standard practice to average time-series data between specified time windows of interest. The windows vary depending on task paradigm, however a common occurrence is to average data before and after odor stimulus onset. Frie et al. (2019) and Onuma and Sakai (2018) use an analysis window of 10 s prior to and 20 post stimulus onset. Hucke et al. (2018) average multiple windows of interest in intervals of 5 s following onset. Other common methodologies involve averaging whole task blocks (Xu et al., 2020). Statistical analysis was conducted for all fNIRS data, predominantly consisting of ANOVAs', *t*-tests and correlation analysis, e.g., Pearson's correlation analysis. In instances of non-parametric data, analysis was generally conducted using Wilcoxon's Test or the Friedman's Test. One paper utilized the General Linear Model to conduct analysis (Gramlich et al., 2017). Seven studies opted to use multiple corrections methods, predominantly Bonferroni's correction, with one using Tukey's and one using Dunnett's methods. If additional corrections were used, they were not stated in the articles. Future research should aim to both use and report multiple corrections. Processing and stats are summarized in Figure 14.

This review highlights the relatively low presence of odor fNIRS studies that exist in this field, but also the potential and applicability of fNIRS to odor. The limited availability of research currently in this area may be due to a lack of standardized protocols with regards to odor stimuli, or the wildly variable individual experiences with odors, which is known to have an impact on both brain and behaviour. In these instances, results may be analyzed in conjunction with questionnaires rating hedonic fragrance properties such as “liking” or “dislike,” as has been done with some studies found in this review.

With regards to standardized protocols, the results of this review suggest that passive and more natural inhalation is preferred over the use of olfactometers. This in combination with fNIRS, and in particular, wearable fNIRS devices, could allow for a diverse range of in-context olfactory studies in the future, e.g., fNIRS during a forest walk, whilst cooking, browsing a perfume isle. Some research has been conducted using behavioral measures and EEG to investigate odor properties, providing a knowledge pool from which to carefully select odors based on desired characteristics and expected responses. The use of therapeutic essential oils such as Ylang ylang, Hinoki cypress tree, lavender, and rose have been discussed in a few papers, providing information regarding their odor properties, however more can be done to develop this research area further with fNIRS, including but not limited to significantly larger population sizes and the inclusion of specifically chosen odors based on known properties or odor families. Studies may wish to investigate the properties of particular odors on sleep quality and duration with portable fNIRS, as is being investigated on a larger scale with EEG in recent years (Chandharakool et al., 2020; Ko et al., 2021; Kwangjai et al., 2021). There is potential here for invigorating odors to be used as a counter to fatigue and sleep onset during driving, to promote cognitive performance under sleep deprived conditions, and for the sleep delaying efficacy of specific odors to be tested using this established paradigm. Similarly, the invigorating effects of some essential oils may be utilized in situations requiring enhanced and prolonged attention, such as during study or in school. The diversity of odor in daily life leads there to be many avenues of study possible with the use of fNIRS, in particular, wearable systems.

## Conclusion

To finalize the results of this review, the presented studies have been summarized to identify common characteristics and findings between studies, in addition to areas to improve for future odor research. In answer to the queries of this article, due to the interconnectivity of odor within the brain, fNIRS is able to measure cerebral changes in oxygenation in response to odors despite having limited depth penetration. The ability to use fNIRS in odor research rather than the static and costly alternative of fMRI, provides immense scope for odor research in the future, which increases further with the use of wearable fNIRS devices. The ability to move freely and unconfined without risk of significant signal loss is a unique asset of fNIRS that has the potential to expand odor research into a multifaceted and vibrant field, encompassing elements of daily life that cannot be explored within laboratory settings, or in confined paradigms involving fMRI or EEG. The use of fNIRS in odor in wellbeing research is address in some studies within this review. There is significant potential here for odor, and in particular, essential oils, to be used to promote wellbeing through sleep and relaxation. The various paradigms presented here involving odor may be adapted or replicated in the future to utilize specific essential oils and wearable fNIRS systems more effectively, with the aim of increasing ecological validity. Short channels and physiological measurements were not commonly used in the studies presented, however, if available can provide an additional method of removing physiological systemics in future studies and understanding the effects of odor further. In this particular field, the strengths of this modality over its counterparts are evidently clear. With the continual development of new fNIRS technologies as well as standardized protocols, the scope for study will only continue to increase, bringing the ambition of improved odour understanding and general wellbeing for all ever closer.

## Data availability statement

The raw data supporting the conclusions of this article will be made available by the authors, without undue reservation.

## Author contributions

NG, IT, and GG contributed to the conception of this work. NG completed all data collection, analysis, and writing and editing. IT, GG, AL, and EB contributed toward manuscript feedback and improvement. IT approved the submitted version of the manuscript. All authors contributed to the article and approved the submitted version.
